# Recognizing emotions induced by wearable haptic vibration using noninvasive electroencephalogram

**DOI:** 10.3389/fnins.2023.1219553

**Published:** 2023-07-06

**Authors:** Xin Wang, Baoguo Xu, Wenbin Zhang, Jiajin Wang, Leying Deng, Jingyu Ping, Cong Hu, Huijun Li

**Affiliations:** ^1^The State Key Laboratory of Digital Medical Engineering, Jiangsu Key Laboratory of Remote Measurement and Control, School of Instrument Science and Engineering, Southeast University, Nanjing, China; ^2^Guangxi Key Laboratory of Automatic Detecting Technology and Instruments, Guilin University of Electronic Technology, Guilin, China

**Keywords:** affective haptics, wearable haptic vibration, electroencephalogram, affective computing, emotion recognition

## Abstract

The integration of haptic technology into affective computing has led to a new field known as affective haptics. Nonetheless, the mechanism underlying the interaction between haptics and emotions remains unclear. In this paper, we proposed a novel haptic pattern with adaptive vibration intensity and rhythm according to the volume, and applied it into the emotional experiment paradigm. To verify its superiority, the proposed haptic pattern was compared with an existing haptic pattern by combining them with conventional visual–auditory stimuli to induce emotions (joy, sadness, fear, and neutral), and the subjects’ EEG signals were collected simultaneously. The features of power spectral density (PSD), differential entropy (DE), differential asymmetry (DASM), and differential caudality (DCAU) were extracted, and the support vector machine (SVM) was utilized to recognize four target emotions. The results demonstrated that haptic stimuli enhanced the activity of the lateral temporal and prefrontal areas of the emotion-related brain regions. Moreover, the classification accuracy of the existing constant haptic pattern and the proposed adaptive haptic pattern increased by 7.71 and 8.60%, respectively. These findings indicate that flexible and varied haptic patterns can enhance immersion and fully stimulate target emotions, which are of great importance for wearable haptic interfaces and emotion communication through haptics.

## 1. Introduction

Emotions play a crucial role in human social communication and interaction (Keltner et al., 2019). With the development of computer technology and human-computer interaction, the field of affective computing (Picard, 1997) has emerged with the primary objective of studying and developing theories, methods, and systems to recognize, interpret, process, and simulate human emotions. Emotions normally change in response to external stimuli, and haptic stimuli can convey more intricate and subtle emotional experiences to the human body compared to visual and auditory stimuli (Hertenstein et al., 2006, 2009). Consequently, a new research trend has arisen in affective computing, which aims to explore the potential of incorporating haptic technology into the processes of emotion recognition, interpretation, and simulation. This integration of haptic technology with affective computing has given rise to a new area called “Affective Haptics” (Eid and Al Osman, 2016).

Affective haptics focuses on the analysis, design, and evaluation of systems capable of inducing, processing, and simulating emotions through touch, which has been applied in many fields. For example, e-learning applications may benefit from affective haptics by reinvigorating learners’ interest when they feel bored, frustrated, or angry (Huang et al., 2010). In healthcare applications, affective haptics can be used to treat depression and anxiety (Bonanni and Vaucelle, 2006), as well as assist in the design of enhanced communication systems for children with autism (Changeon et al., 2012). Other applications include entertainment and games (Hossain et al., 2011), social and interpersonal communication (Eid et al., 2008), and psychological testing (Fletcher et al., 2005).

Affective haptics consists of two subfields: emotion recognition and haptic interfaces. Emotion recognition is to identify emotional states through the user’s behavior and physiological reactions (Kim et al., 2013). Haptic interfaces provide a communication medium between touch and the human subject (Culbertson et al., 2018). The current status of these two subfields will be discussed in the following paragraphs.

Emotion recognition can be broadly divided into two categories: recognition based on non-physiological signals and physiological signals (Fu et al., 2022). Non-physiological signals, such as speech signals (Zhang et al., 2022), facial expressions (Casaccia et al., 2021), and body posture (Dael et al., 2012), are easily influenced by personal volition and the environment, which makes it hard to accurately evaluate an individual’s emotional state. Conversely, physiological signals, which include electroencephalogram (EEG) (Li et al., 2018), electrocardiogram (ECG) (Sarkar and Etemad, 2022), electromyogram (EMG) (Xu et al., 2023), and electrodermal activity (EDA) (Yin et al., 2022), vary according to emotional states, thus providing a more objective means of measuring emotions (Giannakakis et al., 2022). Among these signals, EEG is widely applied in various fields (Zhang et al., 2023; Zhong et al., 2023) and particularly closely associated with emotions (Zhang et al., 2020), so emotion recognition based on EEG has gained widespread usage.

The process of EEG-based emotion recognition involves emotion induction, EEG data preprocessing, feature extraction, and classification models. Koelstra created a publicly available emotion dataset, the DEAP dataset, which contains the EEG and peripheral physiological signals of subjects when watching music videos (Koelstra et al., 2012). Additionally, Zheng also published continuously three emotion datasets based on 62-channel EEG signals: SEED, SEED-IV, and SEED-V (Zheng et al., 2019a,b; Wu et al., 2022). Currently, the above datasets are commonly utilized to extract various features and boost the classification performance by deep learning algorithms (Craik et al., 2019). Liu proposed a three-dimension convolution attention neural network composed of spatio-temporal feature extraction module and EEG channel attention weight learning module (Liu et al., 2022). Zhong proposed a regularized graph neural network for EEG-based emotion recognition and validated its superiority on two public datasets, SEED, and SEED-IV (Zhong et al., 2022). The datasets mentioned above induced emotions in subjects using movie clips of specific emotions. Recently, a few researchers attempted to combine other senses to induce emotions. Wu developed a novel experimental paradigm that allowed odor stimuli to participate in video-induced emotions, and investigated the effects of the different stages of olfactory stimuli application on subjects’ emotions (Wu et al., 2023). Raheel verified that enhancing more than two of the human senses from cold air, hot air, olfaction, and haptic effects could evoke significantly different emotions (Raheel et al., 2020). In general, emotion induction often relies on visual–auditory stimuli, whereas research on emotions induced by haptic stimuli remains quite limited. In other words, there are few studies on recognizing emotions using EEG signals in affective haptics, which to some extent hinders the development of this field.

The haptic interfaces in affective haptics are primarily used to transmit touch sensations to the user through haptic devices (Culbertson et al., 2018). Incorporating haptic devices into emotional induction can convey feelings that are difficult to express with visual–auditory stimuli. Nardelli developed a haptic device that mimicked the sensation of stroking by moving a fabric strip at varying speeds and pressures to examine how the speed and pressure of haptic stimuli elicit different emotional responses (Nardelli et al., 2020). Tsalamlal used a haptic stimulation method of spraying air on the participant’s arm (Tsalamlal et al., 2018). Haynes developed a wearable electronic emotional trigger device that produced a sense of pleasure by stretching and compressing the skin surface of the wearer (Haynes et al., 2019). Wearable devices such as haptic jackets (Rahman et al., 2010) and haptic gloves (Mazzoni and Bryan-Kinns, 2015) are also commonly used in the field of affective haptics. Ceballos designed a haptic jacket and proposed a haptic vibration pattern that enhanced emotions in terms of valence and arousal (Ceballos et al., 2018). Subsequently, Li combined this vibration pattern with visual–auditory stimuli to form a visual–auditory-haptic fusion induction method, demonstrating that haptic vibration improved the accuracy of EEG-based emotion recognition tasks (Li et al., 2022). Whereas, the design of haptic patterns requires further exploration to better understand the mechanism between haptics and emotions.

In conclusion, significant progress has been made in the field of affective haptics. However, the mechanism between haptics and emotions has not yet been clearly revealed. This is primarily due to: (1) the limited application of objective emotion recognition methods in affective haptics; and (2) the lack of diverse haptic patterns. To address these issues, this study designed two haptic vibration patterns and combined them with conventional visual–auditory stimuli to induce emotions (joy, sadness, fear, and neutral). EEG signals were utilized to classify four target emotions and to explore the effects of haptic stimuli on emotions. The contributions of this work are summarized as follows,

- This paper proposed a novel haptic pattern with adaptive vibration intensity and rhythm according to the video volume, and applied it into the EEG emotional experiment paradigm.
- Compared to the existing haptic pattern with fixed vibration intensity and rhythm, the proposed haptic pattern significantly enhanced emotions.
- This paper analyzed the possible reasons for emotional enhancement due to haptic vibration from the perspective of neural patterns.

## 2. Materials and methods

**Figure 1:**
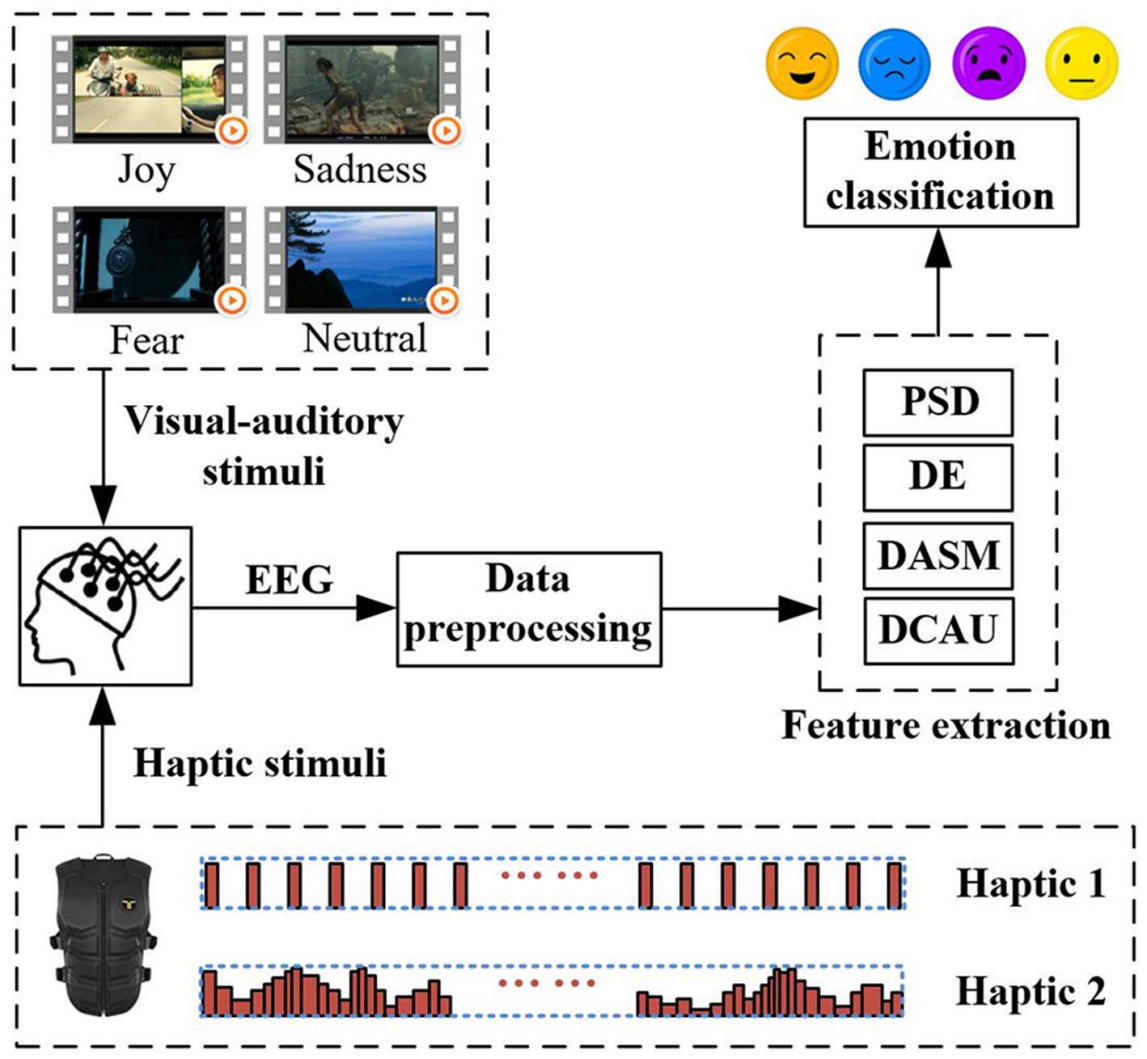
The framework for emotion recognition that incorporates two haptic patterns with traditional visual–auditory stimuli.

In this work, we presented a novel framework for emotion recognition in combination with two haptic vibration patterns, as illustrated in Figure 1. It included visual–auditory-haptic fusion stimuli, EEG acquisition, EEG pre-processing, feature extraction, and emotion classification. Compared to the conventional EEG-based emotion recognition framework, the innovation of this study is to incorporate two haptic patterns with visual–auditory stimuli to explore the effects on emotions. The former is an existing haptic pattern, named Haptic 1. The latter is the proposed adaptive haptic pattern, named Haptic 2. Please see Section 2.3 for details of the two haptic patterns.

### 2.1. Subjects

Sixteen subjects (11 males and 5 females) aged between 20 and 30 years old, all right-handed and with no history of psychiatric illness, participated in the emotion experiment. Prior to the experiment, they were informed about the procedure and allowed to adapt to the experimental setting. The study was approved by the Ethics Committee of Southeast University, and all subjects received compensation for their involvement in the experiment.

### 2.2. Experimental setup

**Figure 2:**
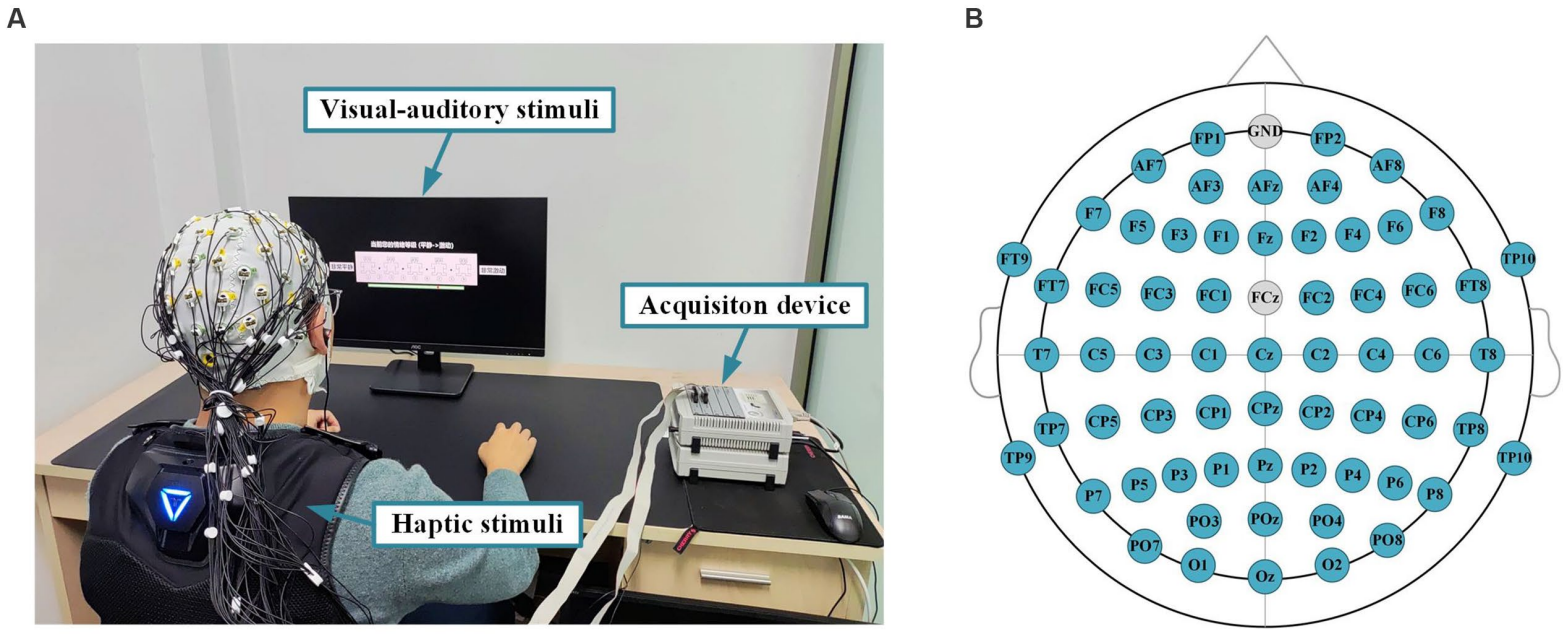
Experimental setup and paradigm for emotion recognition. **(A)** An experimental platform for evoking subjects’ emotions through the visual–auditory-haptic fusion stimulation. **(B)** The EEG cap layout for 64 channels.

In this experiment, the subjects were exposed to visual–auditory-haptic fusion stimuli to induce emotion. The subjects wore a haptic vest and sat in a comfortable chair approximately 0.7 meters away from the monitor, as shown in Figure 2A. EEG signals were recorded by a 64-channel active electrode cap (Brain Products GmbH, Germany), following the international standard 10–20 system. All channels were referenced to the FCz channel, and the Fpz channel was chosen as the ground, as shown in Figure 2B, so a total of 63 channels of EEG data are available. During the recording, the impedance of all channels was kept below 10 kΩ. The EEG sampling frequency was set to 1,000 Hz, and a band-pass filter from 0.05 to 100 Hz was utilized to filter the EEG signals to attenuate high-frequency band components. Meanwhile, a notch filter at 50 Hz was applied to reduce power line interference.

In order to elicit target emotions (joy, sadness, fear, and neutral) in the subjects, we employed a team of eight psychology graduate students to jointly select 16 movie clips that characterized the above four emotions. Each emotion corresponded to 4 movie clips, and all the movie clips were accompanied by Chinese subtitles. Further details can be found in Table 1.

**Table 1:** Details of selected film clips.

No.	Label	File clips sources	Film time
1	Joy	Lost in Thailand	1:03:41–1:08:30
2	Joy	Mr. Bean’s Holiday	0:36:47–0:40:55
3	Joy	Kung Fu Hustle	0:31:58–0:36:49
4	Joy	Fight Back to School	0:39:52–0:43:34
5	Sadness	Aftershock	0:19:36–0:23:15
6	Sadness	Aftershock	1:47:49–1:51:47
7	Sadness	To live	1:12:55–1:17:45
8	Sadness	To live	1:59:49–2:03:32
9	Fear	The Conjuring	0:37:06–0:40:41
10	Fear	The Skeleton Key	1:23:43–1:27:29
11	Fear	Black Swan	1:21:48–1:25:20
12	Fear	Dead Silence	0:08:09–0:12:11
13	Neutral	Huangshan documentary	0:00:50–0:04:36
14	Neutral	Mount Tai documentary	0:00:38–0:04:36
15	Neutral	Aerial China: Xinjiang	0:2:29–0:6:33
16	Neutral	Aerial China: Shanxi	0:31:05–0:34:53

**Figure 3:**
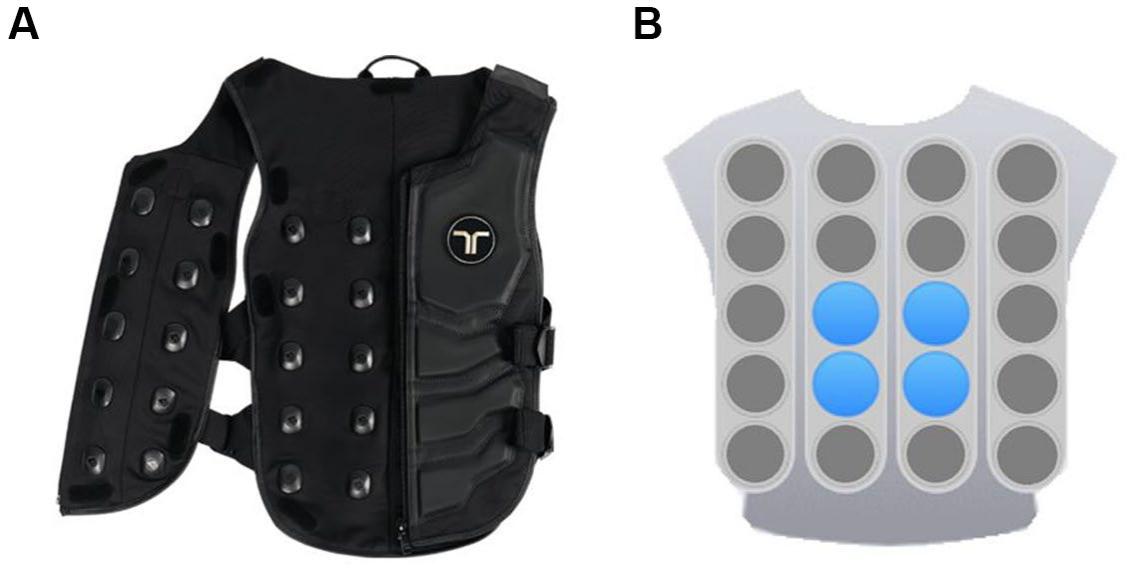
The haptic vest with a dual vibration motors matrix: **(A)** Vest. **(B)** Front view of the motors matrix.

The haptic stimuli implemented in the experiment were realized by using a haptic vest from bHaptics Inc., as illustrated in Figure 3A. As we can see from Figure 3B, this wearable and portable vest provides a double 5 × 4 matrix with motors positioned at both the front and back areas. Each motor provides two methods of vibration modulation: the first is to set the motor’s vibration intensity and rhythm directly, while the second one adjusts the intensity and rhythm of the motor adaptively according to the intensity and frequency of the audio signals. Various haptic vibration patterns can be created by setting the individual parameters of each motor and the overall linkage to give users a specific feeling. The device is controlled through the Unity application via Bluetooth. By incorporating corresponding haptic vibration patterns with different movie clips, we generated emotional stimulation materials that combine visual, auditory, and haptic sensations. The detailed descriptions of the vibration patterns are provided in the next section Experimental protocol.

### 2.3. Experimental protocol

**Figure 4:**
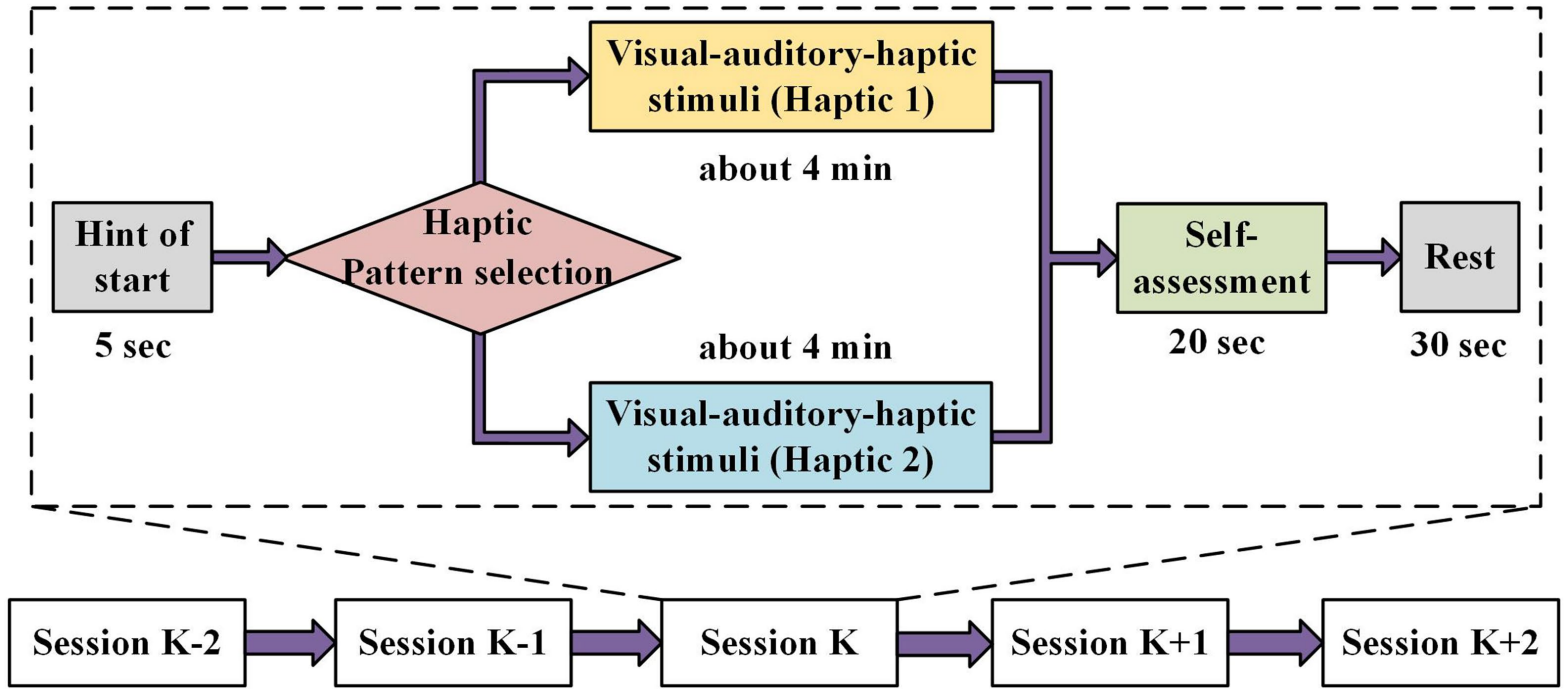
Emotion experimental paradigm based on the visual–auditory-haptic stimuli. The visual–auditory stimuli include a previously selected film clip and the haptic stimuli chosen from either of the two haptic patterns.

Two different haptic vibration patterns were designed to explore their differential effects on emotion. The detailed flow of the emotion experiment is depicted in Figure 4. In total, there were 16 sessions for each experiment. Firstly, each session had a 5-s cue to start. Next, the visual–auditory-haptic fusion stimuli were applied for approximately 4 min, where the visual–auditory stimuli used a previously selected film clip and the haptic stimuli were chosen from either of the two haptic patterns. Then, the subjects were required to conduct a 20-s self-assessment, followed by a 30-s rest. During the self-assessment, the subjects were requested to declare their emotional responses to each session, which would be used later as a reference for assessing the validity of the collected data.

**Figure 5:**
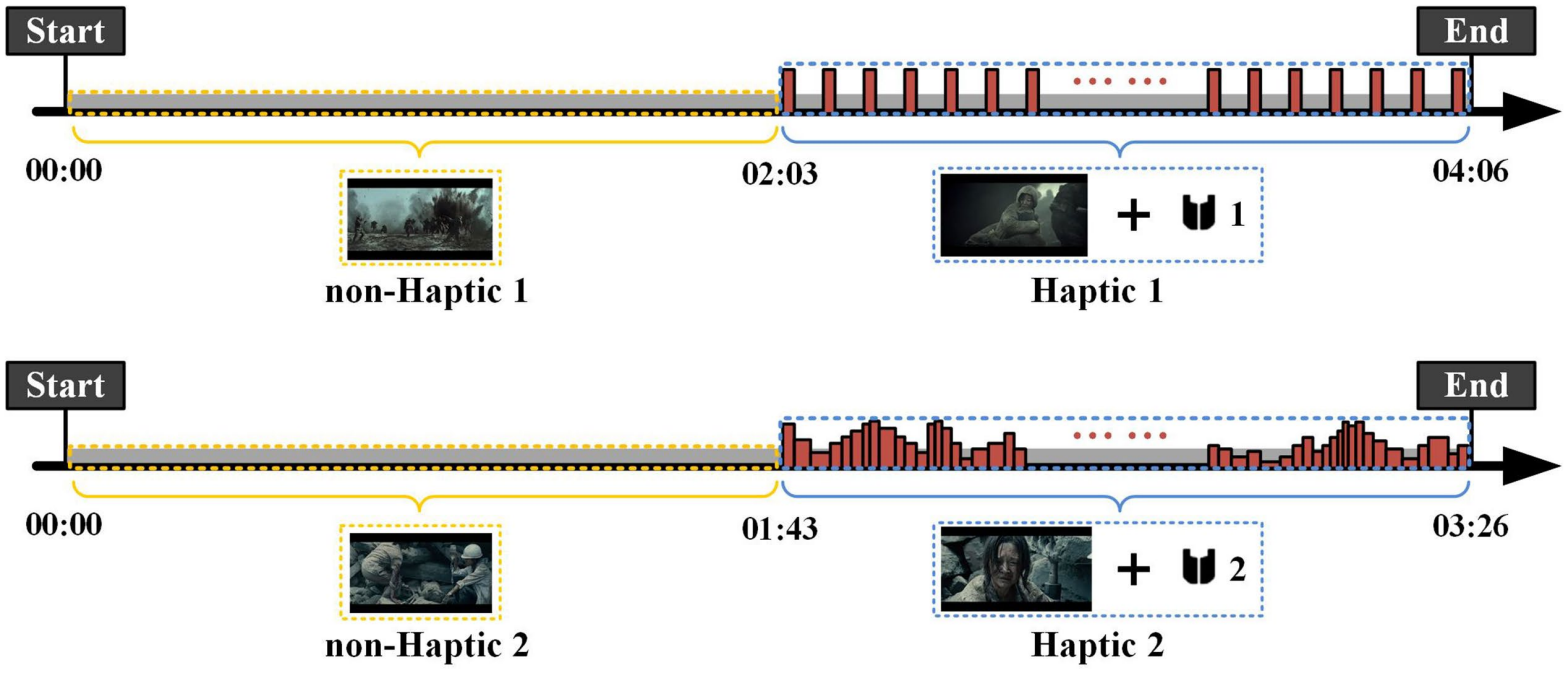
Procedure of two visual–auditory-haptic fusion stimuli. The visual–auditory stimuli were presented as movie clips throughout the experiment, while the haptic stimuli were applied only in the second half of each clip. The first haptic vibration pattern employed a fixed vibration intensity and rhythm, and the second haptic pattern adapted the vibration intensity and rhythm to the video volume.

The visual–auditory-haptic fusion stimuli scheme is shown in Figure 5. The visual–auditory stimuli were presented as movie clips throughout the experiment, while the haptic stimuli were applied only in the second half of each clip to explore whether haptic stimuli could enhance emotions. Importantly, we aimed to examine the differences between the two haptic patterns in inducing emotions. We randomly assigned Haptic 1 or Haptic 2 to the 16 movie clips. The first haptic vibration pattern employed a fixed vibration intensity and rhythm, where each emotion (joy, sadness, and fear) corresponded to a specific intensity and rhythm of vibration, as displayed in Table 2. This pattern has been demonstrated to be effective in previous studies (Ceballos et al., 2018; Li et al., 2022). The second haptic pattern adapted the vibration intensity and rhythm to the video volume. Specifically, the vibration intensity was positively correlated with volume, and the vibration rhythm was adjusted by setting a volume threshold below which no vibration was generated. To maintain sample balance, both patterns were presented for eight sessions.

**Table 2:** Parameters of Haptic 1.

Emotion type	Haptic parameters	
Joy	Frequency: 1.4 Hz Intensity: 90%	Direction: Inward Pattern: Discrete
Sadness	Frequency: 0.45 Hz Intensity: 50%	Direction: Outward Pattern: Discrete
Fear	Frequency: 0.5 Hz Intensity: 90%	Direction: Inward Pattern: Discrete

### 2.4. Data preprocessing

The EEG signals were decomposed using the EEGLAB toolbox. Initially, the sampling rate of the EEG signals was reduced from 1,000 to 200 Hz to expedite computation. Furthermore, a bandpass filter ranging from 1 to 50 Hz was applied to the signals. Then, the independent component analysis (ICA) was employed to remove EOG and EMG artifacts. Furthermore, the common average reference (CAR) was used to re-reference EEG signals to eliminate the global background activity. To correct for stimulus-unrelated variations in power over time, the EEG signal from the 5 s before each video was extracted as a baseline. Finally, the non-haptic and haptic signals were separately intercepted for subsequent analysis. In this study, the EEG signals were divided into five frequency bands: delta (1–4 Hz), theta (4–8 Hz), alpha (8–14 Hz), beta (14–31 Hz), and gamma (31–50 Hz).

### 2.5. Feature extraction and classification

After data preprocessing, we extracted the frequency domain features and their combinations in this study. Four features that proved to be efficient for EEG-based emotion recognition were compared (Zheng et al., 2019b), including PSD, DE, DASM, and DCAU.

The PSD feature is the average energy of EEG signals in five frequency bands for 63 channels, and can be computed directly using a 256-point short-time fourier transform (STFT) with a 1-s-long window and non-overlapped Hanning window. The DE feature is defined as follows,


(1) $$\begin{array}{l} h\left(X\right)=-\overset{\infty }{\underset{-\infty }{\int }}\frac{1}{\sqrt{2\pi \delta^{2}}}\;\exp\frac{{\left(x-\mu \right)}^{2}}{2\delta^{2}}\log\frac{1}{\sqrt{2\pi \delta^{2}}}\\ \;\exp\frac{{\left(x-\mu \right)}^{2}}{2\delta^{2}}dx\;=\;\frac{1}{2}\log2\pi e\delta^{2} \end{array}$$

where the time series *X* obeys the Gauss distribution *N*(*μ*, *δ*^2^), *x* is a variable, and π and *e* are constants. It has been proven that, in a certain band, DE corresponds to the logarithmic spectral energy of a fixed-length EEG series (Shi et al., 2013). Compared with the PSD, DE has a balanced ability to distinguish between low and high frequency energy in EEG patterns.

The DASM feature is calculated as the differences between DE features of 28 pairs of hemispheric asymmetry electrodes (Fp1-Fp2, F7-F8, F3-F4, FT7-FT8, FC3-FC4, T7-T8, P7-P8, C3-C4, TP7-TP8, CP3-CP4, P3-P4, O1-O2, AF3-AF4, F5-F6, FC5-FC6, FC1-FC2, C5-C6, C1-C2, CP5-CP6, CP1-CP2, AF7-AF8, P5-P6, P1-P2, PO7-PO8, PO3-PO4, FT9-FT10, TP9-TP10, and F1-F2), expressed as


(2) $$DASM=h\left(X_{i}^{left}\right)-h\left(X_{i}^{right}\right)$$

The DCAU feature is defined as the differences between DE features of 22 pairs of frontal-posterior electrodes (FT7-TP7, FC5-CP5, FC3-CP3, FC1-CP1, FCZ-CPZ, FC2-CP2, FC4-CP4, FC6-CP6, FT8-TP8, F7-P7, F5-P5, F3-P3, F1-P1, FZ-PZ, F2-P2, F4-P4, F6-P6, F8-P8, Fp1-O1,Fp2-O2, AF3-CB1, and AF4-CB2). DCAU is defined as


(3) $$DCAU=h\left(X_{i}^{frontal}\right)-h\left(X_{i}^{posterior}\right)$$

After feature extraction, we utilized linear-kernel SVM classifiers for the 4-class classification. For statistical analysis, a 4-fold cross-validation strategy was utilized to evaluate the classification performance.

## 3. Results

### 3.1. Analysis of frequency band energy distribution

**Figure 6:**
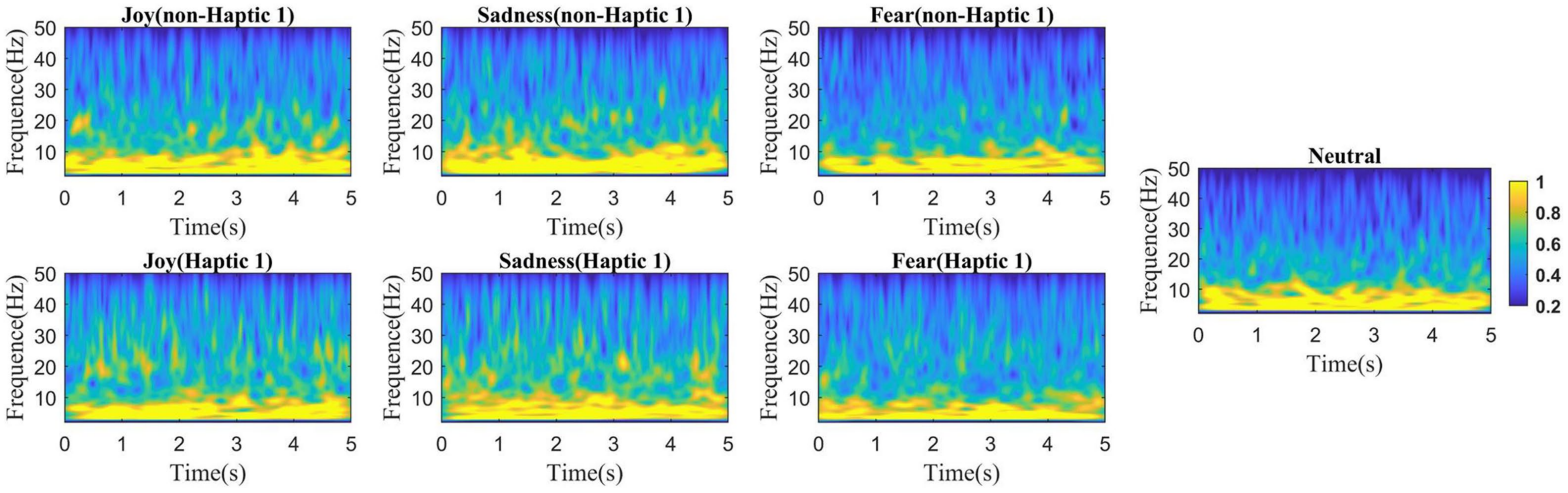
Mean time-frequency analysis based on 16 subjects with non-Haptic 1 or Haptic 1.

**Figure 7:**
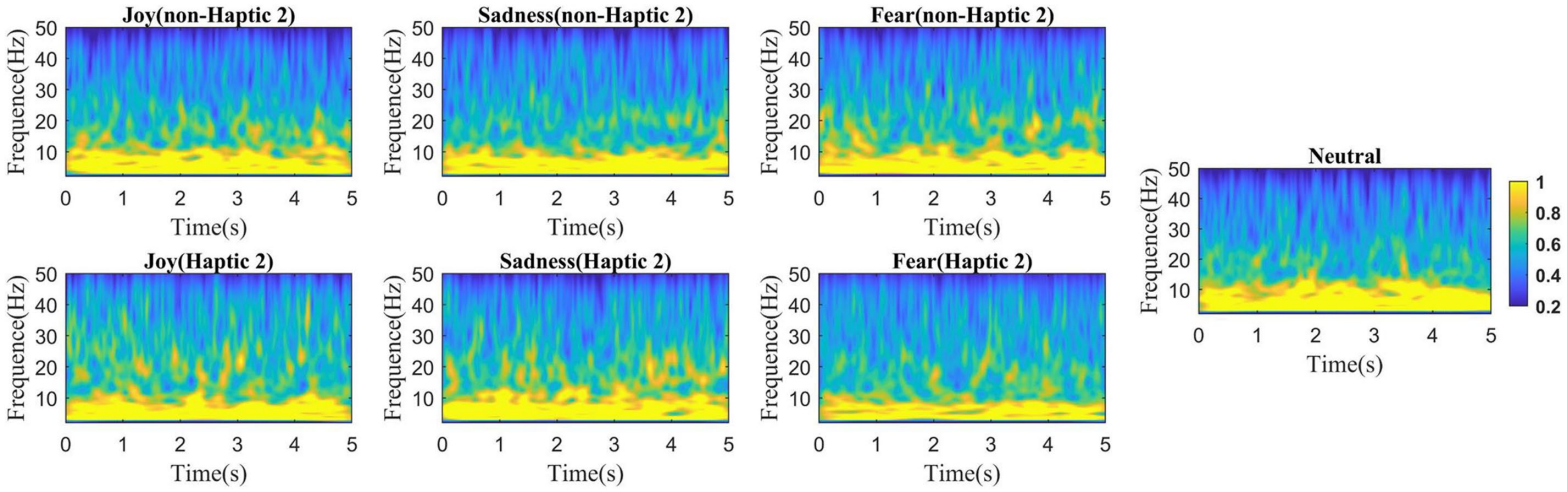
Mean time-frequency analysis based on 16 subjects with non-Haptic 2 or Haptic 2.

To investigate the continual progression of emotional states, we employed wavelet transform methods to conduct time-frequency analysis on EEG signals. A 5-s time window was used to slide the EEG data without overlapping for analysis. According to the two haptic patterns, we calculated the average value of all channels across all subjects in four emotion types, with the results presented in Figures 6, 7. We find that the energy distribution of all emotional states diminishes with an increase in frequency. In comparison to the corresponding non-haptic patterns, the energy distributions of the three emotions (joy, sadness, and fear) are obviously higher in the high-frequency band under the two haptic patterns. Joy and sadness show no obvious changes in the low-frequency band, whereas fear emotions reveal a decrease in energy. This suggests that haptic vibration patterns are capable of affecting various emotions. Moreover, it can be seen that Haptic 2 has more energy in the alpha, beta, and gamma bands than Haptic 1 when comparing Figure 6 with Figure 7. Since the average energy is based on all channels and all time segments, the next paragraph requires further analysis considering topographical maps.

### 3.2. Analysis of brain topographical maps

**Figure 8:**
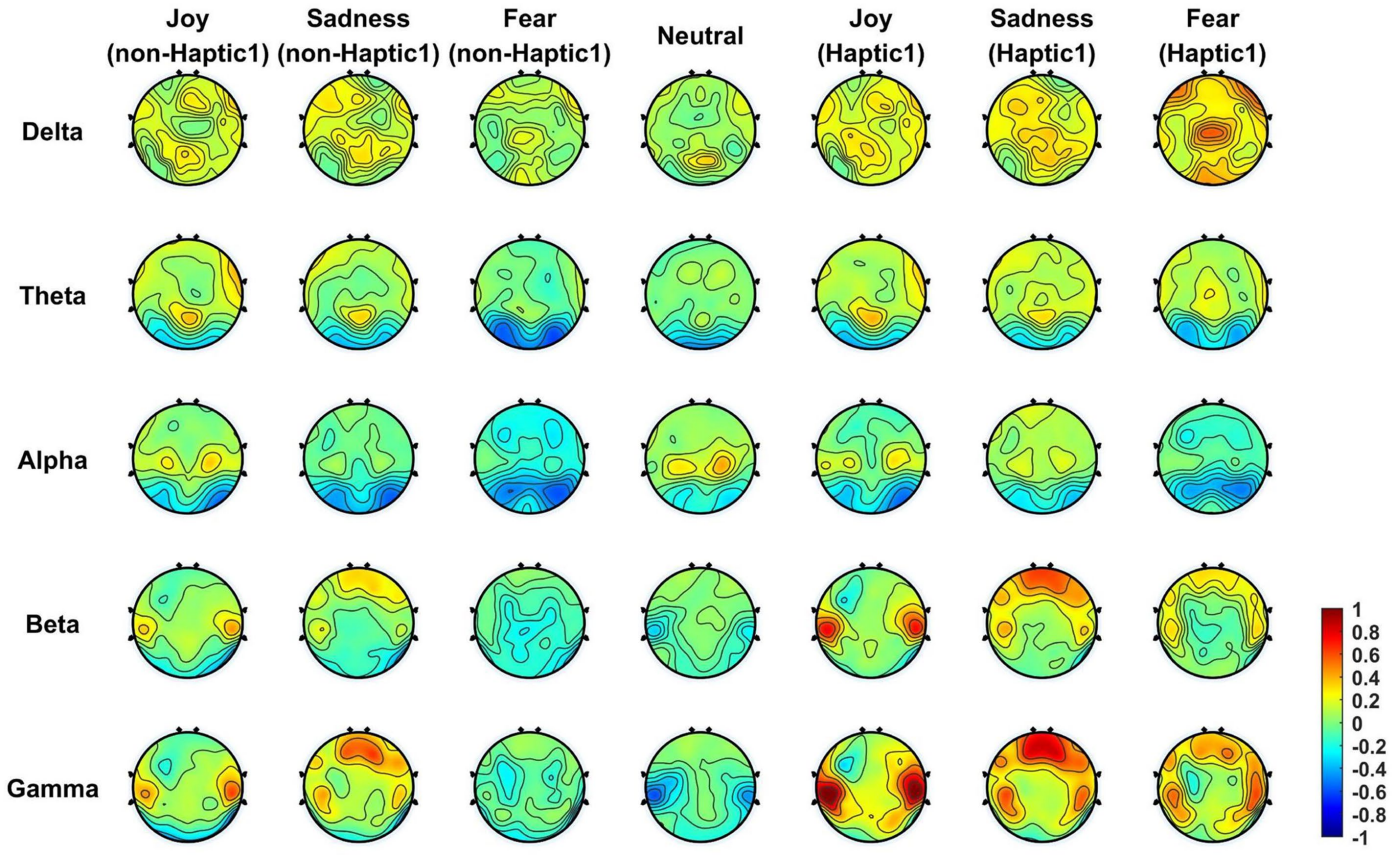
The average neural patterns in different emotional states for 16 subjects with non-Haptic 1 or Haptic 1.

**Figure 9:**
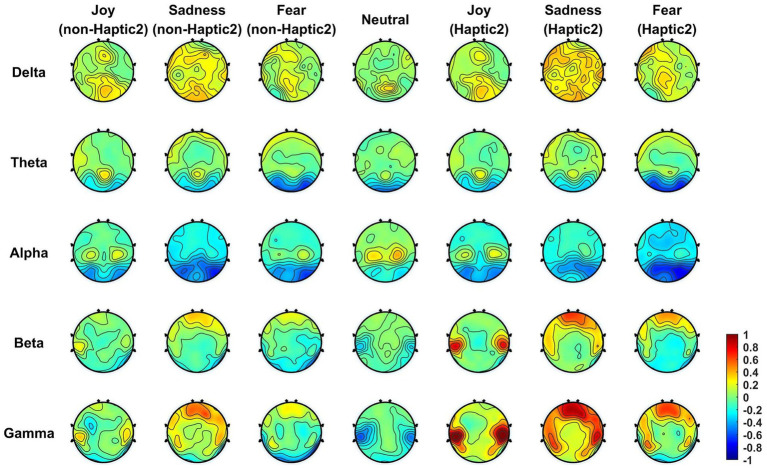
The average neural patterns in different emotional states for 16 subjects with non-Haptic 2 or Haptic 2.

In order to analyze whether and how haptic stimuli affect emotional brain regions, we computed the mean DE features across all subjects in five frequency bands and then projected them onto the scalp. To highlight the changes more distinctly, the corresponding baseline features (collected during the 5-s preparation period) were subtracted from the projected DE features. Figures 8, 9 display the brain topographic maps for different emotional states in non-Haptic 1, Haptic 1, non-Haptic 2, and Haptic 2, respectively.

In Figure 8, the average neural patterns for different emotions in non-Haptic 1 and Haptic 1 are depicted. In the non-Haptic 1 state, the four types of emotions differ in the lateral temporal areas mainly in beta and gamma frequency bands. Specifically, the lateral temporal areas exhibit obvious activation for happy emotions in beta and gamma bands, while the corresponding areas for neutral emotions are inhibited. The prefrontal areas of sadness show more activation in beta and gamma bands compared to other emotions. The alpha band activation in the parietal areas is higher for happy and neutral emotions than for sadness and fear. The occipital regions in the theta and alpha bands for all emotions show low activation, with sadness and fear showing less activation. Afterward, with the application of Haptic 1, the energy distribution of different emotions across brain regions follows a similar trend as non-Haptic 1, but with more notable variations. The lateral temporal areas show more activation in beta and gamma bands for joy. The lateral temporal and parietal areas for sadness and fear concentrate more energy in both beta and gamma bands. Therefore, haptic vibration not only maintains the fundamental neural patterns for different emotions, but also increases the activation of the lateral temporal and prefrontal areas.

Figure 9 presents the average neural patterns of the four emotions for all subjects in non-Haptic 2 or Haptic 2. In non-Haptic 2, the brain topography maps for the four emotions are similar to those displayed in Figure 8, with only minor discrepancies. This phenomenon can be attributed to the fact that subjects are unlikely to respond identically to various audiovisual materials conveying the same emotion. Subsequently, the neural patterns of the different emotions in Haptic 2 state showed some similarity to those of Haptic 1. However, there is a more concentrated distribution of energy in brain regions in Haptic 2 state, with greater activation in lateral temporal and prefrontal areas in beta and gamma bands, and more inhibition in occipital areas in alpha bands. Hence, the above two haptic patterns indeed have different effects on emotion-related regions.

### 3.3. Classification performance

**Figure 10:**
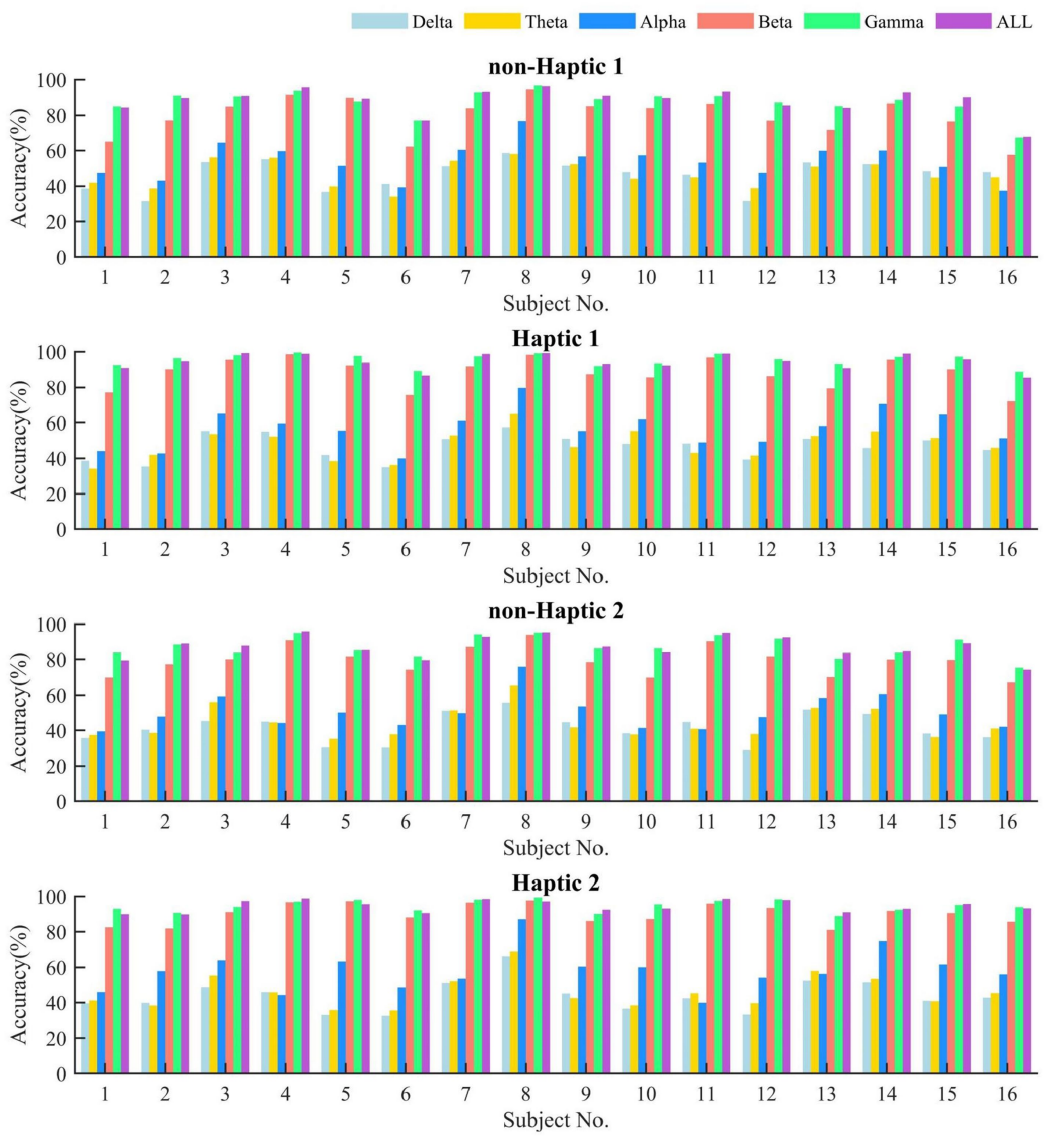
The average classification accuracy of DE feature by SVM in different frequency bands with non-haptic and haptic patterns.

We employed SVM to classify the DE features in all frequency bands. Figure 10 provides a visual representation of the emotional classification results for 16 subjects in non-Haptic 1, Haptic 1, non-Haptic 2, and Haptic 2, respectively. It can be observed that the classification accuracy of DE features in beta, gamma, and full band frequency ranges is significantly higher than in delta and theta bands. This indicates that the delta and theta bands have little impact on emotion recognition, similar to the difficulty of finding obvious differences between these two bands in different emotions in brain topographic maps. Moreover, the accuracy in haptic patterns is significantly higher than that in non-haptic patterns, especially in beta, gamma, and full band frequencies. The results demonstrate that combining traditional visual–auditory stimuli with haptic stimuli can effectively induce emotions.

Tables 3, 4 present the average SVM classification results of different features of the five frequency bands in Haptic 1 and Haptic 2, respectively. Meanwhile, the paired-sample *t*-test results on the accuracy of four features in different frequency bands for the two cases of non-Haptic 1 - Haptic 1, and non-Haptic 2 - Haptic 2 are shown in Table 5. The results indicate that the average accuracy of PSD, DE, DASM, and DCAU features in the two haptic patterns is significantly higher than that in non-haptic patterns, demonstrating that haptic stimuli can enhance subjects’ emotions. In most cases, the average accuracy of DE features is higher than that of the other three features, indicating that DE features are superior in representing emotions. Additionally, the average accuracy of beta and gamma bands is significantly higher than that of other bands for four features. These findings suggest that beta and gamma bands play a crucial role in EEG-based emotion recognition and are highly correlated with emotional states. These quantitative results are consistent with the qualitative results obtained from the brain topographic maps.

**Table 3:** The average classification accuracy of four features in different frequency bands with non-Haptic 1 and Haptic 1.

	PSD		DE		DASM		DCAU	
	Non-Haptic 1	Haptic 1	Non-Haptic 1	Haptic 1	Non-Haptic 1	Haptic 1	Non-Haptic 1	Haptic 1
Delta	40.77%	40.96%	46.60%	46.73%	36.36%	36.40%	37.99%	38.27%
Theta	40.12%	40.92%	46.99%	47.87%	35.70%	35.85%	38.58%	38.75%
Alpha	45.37%	47.72%	54.05%	56.75%	39.81%	41.48%	40.89%	42.18%
Beta	72.47%	82.06%	79.60%	88.35%	65.37%	73.36%	60.59%	68.58%
Gamma	82.56%	91.16%	87.30%	95.42%	77.06%	84.11%	70.15%	79.37%
Total	82.82%	89.87%	88.09%	94.53%	78.99%	85.69%	75.34%	82.62%

**Table 4:** The average classification accuracy of four features in different frequency bands with non-Haptic 2 and Haptic 2.

	PSD		DE		DASM		DCAU	
	Non-Haptic 2	Haptic 2	Non-Haptic 2	Haptic 2	Non-Haptic 2	Haptic 2	Non-Haptic 2	Haptic 2
Delta	37.28%	38.16%	41.64%	43.90%	33.60%	34.59%	35.52%	35.81%
Theta	38.27%	39.39%	44.19%	46.01%	34.39%	35.24%	36.88%	38.07%
Alpha	42.59%	49.54%	50.19%	57.96%	38.77%	43.40%	39.09%	42.41%
Beta	72.05%	84.61%	79.52%	90.17%	66.06%	76.35%	57.09%	70.86%
Gamma	82.41%	91.79%	87.34%	94.60%	77.89%	86.32%	68.49%	79.56%
Total	80.81%	91.40%	87.31%	94.51%	78.32%	86.79%	71.59%	82.36%

**Table 5:** Paired-sample *t*-test results on the accuracy of four features in different frequency bands for two cases of non-Haptic 1 - Haptic 1, and non-Haptic 2 - Haptic 2 (*α* = 0.05).

	(Non-Haptic 1) – (Haptic 1)				(Non-Haptic 2) - (Haptic 2)			
	PSD	DE	DASM	DCAU	PSD	DE	DASM	DCAU
Delta	0.7921	0.8896	0.9598	0.5535	0.3148	0.0138	0.0624	0.7836
Theta	0.8374	0.4872	0.0686	0.1751	0.0469	0.0045	0.2201	0.2252
Alpha	0.1325	0.0745	0.2065	0.4003	0.0001	0.0001	0.0001	0.0286
Beta	0.0001	0.0001	0.0001	0.0001	0.0001	0.0001	0.0001	0.0001
Gamma	0.0001	0.0001	0.0001	0.0001	0.0001	0.0001	0.0001	0.0001
Total	0.0001	0.0001	0.0001	0.0001	0.0001	0.0001	0.0001	0.0001

To further compare the emotional enhancement effects of different haptic patterns, we calculated the average classification accuracy growth rates of four features across various frequency bands in Haptic 1 and Haptic 2, as presented in Table 6. It can be seen that the accuracy growth rates in Haptic 2 are higher than those in Haptic 1 for all features and all frequency bands, with a more significant increase observed in the alpha and beta frequency bands. Taking DE features as an example, the classification accuracy of Haptic 1 and Haptic 2 increased by 7.71 and 8.60%, respectively. In particular, as shown in Table 5, Haptic 2 presents a significant improvement in classification accuracy compared to not applying haptic stimuli in almost all bands, but the classification accuracy of Haptic 1 is not significantly improved in the lower bands. These results suggest that Haptic 2, with adaptive vibration intensity and rhythm, is more effective in eliciting emotions than Haptic 1, which has constant vibration intensity and rhythm.

**Table 6:** The average classification accuracy growth rates of four features in different frequency bands with Haptic 1 and Haptic 2.

	PSD		DE		DASM		DCAU	
	Growth rate 1	Growth rate 2	Growth rate 1	Growth rate 2	Growth rate 1	Growth rate 2	Growth rate 1	Growth rate 2
Delta	0.85%	2.12%	1.23%	5.76%	0.63%	3.32%	0.33%	0.11%
Theta	0.24%	3.05%	2.09%	4.13%	0.52%	2.37%	0.93%	3.60%
Alpha	5.67%	17.36%	5.57%	15.87%	4.89%	12.94%	3.69%	9.70%
Beta	14.36%	18.22%	11.75%	13.93%	13.56%	16.04%	14.55%	25.23%
Gamma	11.35%	11.95%	7.79%	8.62%	9.93%	11.40%	14.36%	16.96%
Total	8.95%	13.66%	7.71%	8.60%	9.49%	11.46%	10.35%	15.56%

## 4. Discussion

### 4.1. Important brain regions and frequency bands for emotion

This paper presented the average neural patterns associated with four emotional states evoked by visual–auditory stimuli, as depicted in Figures 8, 9. Notably, the activation of lateral temporal and prefrontal areas in beta and gamma bands varied obviously in different emotional states. It suggests that these brain regions are highly correlated with different emotions and are considered the key regions for generating emotions. Overall, these findings are consistent with the results of previous research (Zheng and Lu, 2015; Zheng et al., 2019b). Besides, the accuracy of emotion classification was obviously higher in beta and gamma bands compared to other single bands. Interestingly, in some cases, the accuracy in total bands was lower than that in gamma bands. This may be due to the low classification accuracy of lower frequency band signals, which disturb the overall emotion classification. In summary, collecting EEG signals in beta and gamma bands from lateral temporal and prefrontal regions is an effective approach for recognizing emotions induced by visual–auditory stimuli. This finding can be utilized as a reference to simplify the number of EEG acquisition electrodes and reduce the data scale.

### 4.2. Neural pattern for haptic-enhanced emotion

This paper demonstrated the superiority of the application of haptic patterns over non-haptic patterns in EEG-based emotion recognition. Certainly, previous studies have come to a similar conclusion that haptic stimuli improve the efficiency of emotion recognition tasks (Raheel et al., 2020; Li et al., 2022). However, few studies have analyzed and explained the phenomenon from the perspective of neural patterns. Notably, haptic stimuli not only maintain the fundamental neural patterns for different emotions, but also increase the activation of lateral temporal and prefrontal areas that closely associated with emotion, as illustrated in Figures 8, 9. We speculate that there are two reasons for this phenomenon: (1) Although haptic stimuli applied to the torso typically activate the somatosensory cortex in the parietal area directly, there was only a weak enhancement of the parietal area in the brain topographic maps. However, the brain is a complex interconnected structure, and it is possible that haptic stimuli affect lateral temporal and frontal regions through the somatosensory cortex. Meanwhile, a converging body of literature has shown that the somatosensory cortex plays an important role in each stage of emotional processing (Kropf et al., 2019; Sel et al., 2020). (2) The application of haptic stimuli increased the subjects’ immersion. Rather than exclusively focusing on haptic stimuli, the subjects’ senses may have been fully engaged in watching emotional movie clips.

### 4.3. Efficiency of the proposed haptic pattern

In this paper, a novel haptic pattern (Haptic 2) was designed and compared with the existing haptic pattern (Haptic 1) in EEG emotional paradigm. The experimental results demonstrate that different haptic patterns have varying levels of emotional enhancement. According to the *t*-test in Table 5, the classification accuracy of Haptic 2 was significantly increased over non-haptic pattern in almost all frequency bands. However, the classification accuracy of Haptic 1 was not significantly improved in the lower bands. Furthermore, as shown in Table 6, the classification accuracy growth rates in Haptic 2 were slightly higher than those in Haptic 1. As we can see from Figure 9, Haptic 2 resulted in a more concentrated energy distribution in subjects’ brain regions. Specifically, lateral temporal and prefrontal regions increased activation in beta and gamma bands, while occipital regions exhibited greater inhibition in alpha bands. This amplified difference in energy distribution in emotion-related regions may account for higher classification accuracy growth rates of Haptic 2. Additionally, based on subjective feedback from subjects, most of them said that Haptic 2 was more suitable for the movie scene. We hypothesize that the adaptive adjustment of vibration intensity and rhythm with audio in Haptic 2 can enhance immersion and fully stimulate target emotions compared to Haptic 1. In general, these findings suggest that the proposed haptic pattern has superiority in evoking target emotions to some degree.

### 4.4. Limitations and future work

In our work, we combined two haptic vibration patterns with visual–auditory stimuli to induce emotions and classify four emotions based on EEG signals. However, our study still has certain limitations. Firstly, the number of subjects was not large enough, and the age range was limited to 20 to 30 years old. In the future, we will extend the proposed experiment paradigm to a larger number of subjects and a wider age range to investigate whether there are gender and age differences in the effects of haptic stimuli on emotion. In addition, this study only extracted features from single-channel EEG data, ignoring the functional connectivity between brain regions. Subsequently, we will utilize EEG-based functional connectivity patterns and more advanced deep learning algorithms considering brain topology in future studies. Moreover, our experimental results preliminarily showed the adaptive haptic vibration pattern is more advantageous to enhance emotion, while more detailed and reasonable designs of the haptic patterns require further exploration. In the design of the two haptic patterns, we only considered vibration intensity and rhythm, but neglected the impact of vibration location. Hence, we will create more comprehensive haptic vibration patterns to further investigate the mechanism of haptic stimuli on emotion enhancement.

## 5. Conclusion

The motivation of this study is to investigate the variations in emotional effects induced by different haptic patterns. This paper proposed a novel haptic pattern with adaptive vibration intensity and rhythm according to the video volume, and compared it to the existing haptic pattern in emotional experiment paradigm. Specifically, the above two haptic patterns were combined with traditional visual–auditory stimuli to induce emotions, and four target emotions were classified based on EEG signals. Compared with the visual–auditory stimuli, the visual–auditory-haptic fusion stimuli significantly improved the emotion classification accuracy. The possible reason is that haptic stimuli cause distinct activation in lateral temporal and prefrontal areas of the emotion-related regions. Moreover, different haptic patterns had varying effects on enhancing emotions. The classification accuracy of the existing and the proposed haptic patterns increased by 7.71 and 8.60%, respectively. In addition, the proposed haptic pattern showed a significant improvement in classification accuracy compared to non-haptic pattern in almost all bands. The results show that the haptic pattern with adaptive vibration intensity and rhythm is more effective in enhancing emotion. Therefore, flexible and varied haptic patterns have extensive potential in the field of affective haptics.

## Data availability statement

The raw data supporting the conclusions of this article will be made available by the authors, without undue reservation.

## Ethics statement

The studies involving human participants were reviewed and approved by Ethics Committee of Southeast University. The patients/participants provided their written informed consent to participate in this study.

## Author contributions

XW and BX designed the study, analyzed the data, and wrote the manuscript. XW, WZ, and JW set up the experiment platform. LD and JP performed the experiment. JW, CH, and HL reviewed and edited the manuscript. All authors read and approved the final manuscript.

## Funding

This work was supported by National Key Research and Development Program of China (2022YFC2405602), the Natural Science Foundation of Jiangsu Province (No. BK20221464), the Key Research and Development Program of Jiangsu Province (No. BE2022363), the Basic Research Project of Leading Technology of Jiangsu Province (No. BK20192004), the National Natural Science Foundation of China (Nos. 92148205, 62173088, and 62173089), and Guangxi Key Laboratory of Automatic Detecting Technology and Instruments (No. YQ22207).

## Conflict of interest

The authors declare that the research was conducted in the absence of any commercial or financial relationships that could be construed as a potential conflict of interest.

## Publisher’s note

All claims expressed in this article are solely those of the authors and do not necessarily represent those of their affiliated organizations, or those of the publisher, the editors and the reviewers. Any product that may be evaluated in this article, or claim that may be made by its manufacturer, is not guaranteed or endorsed by the publisher.
